# Theoretical evaluation of Cox’s interaction model of client health behavior for health promotion in adult women

**DOI:** 10.4069/kjwhn.2020.06.13

**Published:** 2020-06-25

**Authors:** Youlim Kim, Hyeonkyeong Lee, Gi Wook Ryu

**Affiliations:** College of Nursing, Mo-Im Kim Nursing Research Institute and College of Nursing, Yonsei University, Seoul, Korea

**Keywords:** Health behavior, Health promotion, Nursing theory, Women

## Abstract

This study aimed to evaluate Cox’s interaction model of client health behavior (IMCHB) as used in studies on women’s health. Using keyword combinations of “women” and “IMCHB” or “interaction model of client health behavior,” we searched the PubMed, MEDLINE, Embase, and RISS databases for studies on the promotion of women’s health published from January 2009 to April 2019. Finally, 11 studies were selected and evaluated according to seven criteria for theory evaluation, which combined Fawcett’s theory evaluation criteria and Chinn and Kramer’s criteria. We found that the IMCHB corresponds to a verifiable practical level of a middle-range theory, although it may be partially abstract. It contains all four concepts of the metaparadigm of nursing, in terms of a holistic philosophical approach. A theoretical evaluation demonstrated that the IMCHB has significance, generality, testability, empirical adequacy, and pragmatic adequacy for nursing practice and research. However, the lack of clear conceptual definitions and the presence of complex relationships among concepts resulted in a lack of internal consistency and parsimony. According to an in-depth verification through a review of the literature, the IMCHB has been used as a health promotion intervention strategy for various populations of women and has led to useful results in nursing practice. The IMCHB was confirmed to be a suitable theory for experimental and clinical research. Future research can build on this middle-range theory for women’s health research and practice.

## Introduction

Women, who make up half of the global population, have unique health needs from childhood to old age. The United Nations emphasized improving women’s health and quality of life in its Sustainable Development Goals [1]. These agendas broadened the concept of women’s social activities and gender equality, and increased the number of studies related to women’s health [2]. In particular, the conceptual focus of women’s health expanded from maternal health, which is focused on pregnancy and childbirth, to include sexual health, depression, drinking, and smoking among women, and is transitioning to encompass gender-centered promotion of women’s health [2]. Due to these changes, researchers in the field of women’s health should consider sociocultural influences, approach the holistic context of life from individuals to motherhood and beyond, and seek to understand phenomena in terms of human interactions [3].

Nursing theory is necessary as a framework for testing various phenomena in the field of nursing in the present rapidly changing social and cultural environment [4], and through such research, scientific knowledge about nursing can be accumulated. Nursing theory provides a conceptual basis for understanding the relationship between theoretical and practical nursing [5].

In order to study women’s health promotion, it is necessary to comprehensively analyze and evaluate relevant theories by considering various aspects of the physical, mental, and social environment of the population [6]. Among the various theories related to health behavior, Cox’s interaction model of client health behavior (IMCHB) includes client-professional interaction as a main concept, highlighting nurses’ professional roles and their provision of patient-centered nursing in consideration of the context of the target population [7]. In addition, Cox’s IMCHB is a nursing model that emphasizes the roles and interactions of clients and professionals and is used as a theoretical framework in various areas of nursing practice and research to contribute to nursing theoretical knowledge [8].

In order to properly explain phenomena encountered in nursing practice, an in-depth critical understanding of a theory is required before applying it. Evaluation of nursing theories can contribute to the consolidation of nursing epistemology and to the further development and improvement of theories [9]. A previous study evaluated the IMCHB using research papers published from 1983 to 1994, the first decade after the theory was developed [9], but no analyses and evaluations of this theory have been conducted for about 25 years thereafter. Therefore, reviewing the research related to women’s health that has applied IMCHB in the last 11 years may suggest future research directions. In this study, as Cox’s IMCHB is a middle-range theory, we reviewed studies that applied the IMCHB to adult women who were not hospitalized for specific diseases.

The purpose of this study was 1) to conduct a theoretical analysis and evaluation of research in the field of women’s health applying Cox’s IMCHB published in domestic and international research journals in the past 11 years (January 2009 to April 2019), and 2) to propose a strategy for nursing theory, research, and practice development for women's health promotion based on this analysis and evaluation.

## Methods

Ethics statement: This study was exempted from the requirement to obtain informed consent because it was a literature review.

### Design and samples

This study was a literature review analyzing papers on women’s health that applied Cox’s IMCHB and were published in domestic and international journals within the past 11 years, from January 2009 to April 2019.

### Data sources

A search was conducted for studies that applied the IMCHB in relation to the health of female outpatients and were published in peer-reviewed journals in Korean or English between January 2009 and April 2019. We searched four online databases: PubMed, MEDLINE, Embase, and the Research Information Sharing Service (RISS). Terms related to the theory were identified by referring to the keywords used in theoretical evaluation papers dealing with the IMCHB [9]. We conducted a preliminary search using the terms “interaction model of client health behavior” or “Cox’s interaction model” in combination with “women.” The preliminary results showed that many articles used the abbreviation “IMCHB” for “interaction model of client health” throughout the text, and it was confirmed that few articles were found when “Cox” was added as a term. Therefore, we used the following final combination of keywords:

((("Female"[Mesh]) OR "(Women"[Mesh]))) AND (("IMCHB") OR ("interaction model of client health behavior")).

### Study selection

The literature selection criteria for this study were: (1) the IMCHB was applied, and (2) the participants were adult women. The criteria for exclusion were: (1) the IMCHB application was not clearly explained, or the participants were (2) inpatients or (3) children and adolescents.

**Figure 1.**
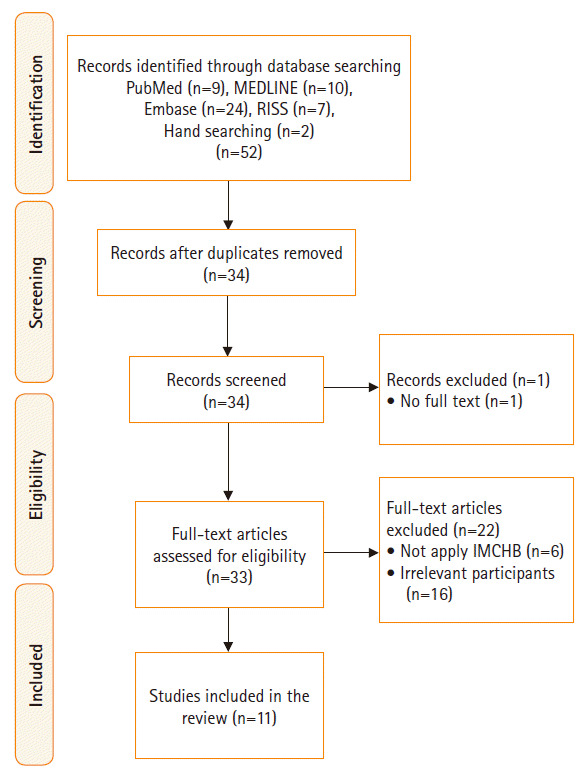
PRISMA flow diagram. IMCHB: Interaction model of client health behavior; RISS: Research Information Sharing Service.

According to the results obtained by searching the international databases using the search terms, 52 studies were found (nine in PubMed, 10 in MEDLINE, 24 in Embase, seven in RISS, and two via hand-searching). After duplicates were removed (n=18), two reviewers (YK and GWR) independently screened a total of 34 articles focusing on titles and abstracts. The same reviewers then independently reviewed the full-text articles to determine whether each article was appropriate for inclusion in this analysis. One study was excluded because the original text was inaccessible and 22 articles met the exclusion criteria (6 studies did not apply the IMCHB, four studies involved inpatients, and 12 studies sampled non-adult women), so 11 studies were included in the analysis (Figure 1).

### Criteria for the evaluation and analysis of theory and literature

In this study, Fawcett’s theory analysis and evaluation steps were used as the main theoretical evaluation criteria [10]. Fawcett [10] presented the analysis and evaluation of theories as involving the stages of “theoretical analysis” and “theoretical evaluation.” Fawcett’s evaluation criteria for theories include significance, internal consistency, parsimony, testability, empirical adequacy, and pragmatic adequacy. Chinn and Kramer [11] proposed different evaluation criteria for theories, and additionally presented generality as a criterion to evaluate theories. Generality refers to assessing whether a theory can explain a wide scope of experiences and phenomena [11]. Therefore, we added the generality criterion of Chinn and Kramer [11] to Fawcett’s evaluation criteria to analyze the IMCHB from various viewpoints. Consequently, in this study, the theory and literature were evaluated according to seven criteria: importance, internal consistency, parsimony, generality, testability, empirical adequacy, and practical adequacy.

## Results

### General characteristics of the reviewed literature

The reviewed studies included five international studies (45.5%) and six studies from South Korea (54.5%) (Table 1). Ten studies (90.9%) were quantitative, and one (9.1%) was qualitative. The participants in the studies were minority groups (immigrants or multicultural women) and low-income women in five studies (45.4%), pregnant women in two studies (18.2%), postpartum women in one study (9.1%), women with gynecological cancer in one study (9.1%), women with osteoarthritis in one study (9.1%), and older adults in one study (9.1%).

### Analysis of the theory

#### Scope: Level of abstraction

The concepts of a theory play a fundamental role in its construction, and the object to which each concept is addressed should be empirically defined [12,13].

Cox’s IMCHB encompasses three major elements: the client background, client-professional interaction, and health outcomes of the client (Table 2). However, these concepts are not clearly explained, and there is no specific statement on how to measure them. As the IMCHB does not address specific health-related situations and was designed to be applied to a wide range of health care decisions and behaviors, it is necessary to keep in mind that the concepts highlighted may vary depending on the research question [14]. Therefore, the IMCHB corresponds to a verifiable practical level of a middle-range theory, and it may be partially abstract due to the lack of concrete descriptions of specific nursing phenomena and clients.

#### Context: Metaparadigm concepts and propositions

To analyze the context of the IMCHB as a theory, it is necessary to ensure that it presents a philosophical opinion based on a metaparadigm and evidence [10].

Cox [7] recognized human beings as independent entities capable of determining their own health behaviors, focused on human singularity, and identified environmental factors as personal factors, economic resources, and use of health care facilities related to background variables. In the IMCHB, “health” is described as positive behaviors obtained through interaction with experts who have recognized the characteristics of the client, and five variables are suggested to measure health outcomes (e.g., health status indicators and healthcare utilization). Nursing interventions involve emotional support, provision of health information, decisional control, and professional/technical competencies through interactions with the client [7].

Cox [15] stated that the IMCHB can be used for the client’s health care based on a holistic philosophy. Therefore, it was confirmed that IMCHB contains all four concepts of the metaparadigm of nursing and views health from a holistic philosophical point of view.

#### Content: Concepts and propositions

**Figure 2.**
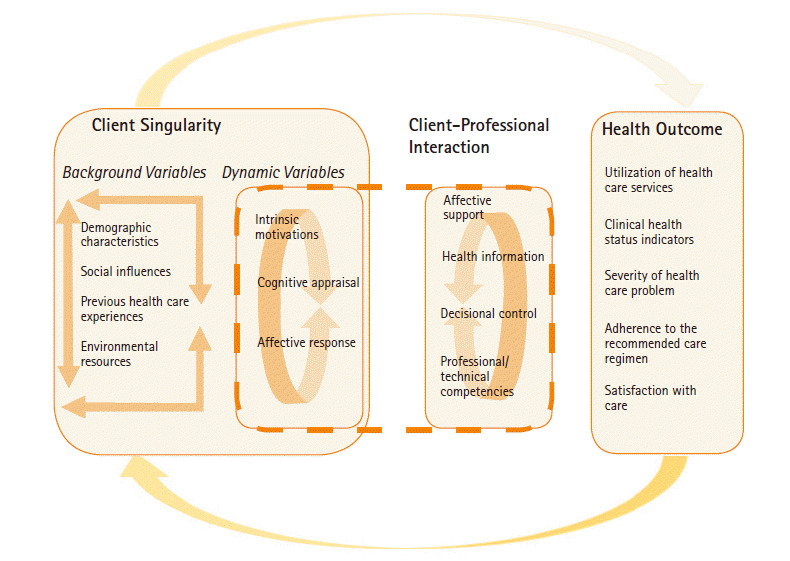
Interaction model of client health behavior. Adapted Figure 1 from the article of Cox et al. [16] (Oncol Nurs Forum 2003;30(5):E92-E99).

The IMCHB is composed of three elements: the singularity of the client, interaction between the client and the professional, and health outcome of the client (Figure 2). It contains seven concepts regarding the client’s singularity, four concepts relating to patient-professional interactions, and five concepts involving the client’s health outcomes (Table 2).

An example drawn from the nine statements that correspond to the IMCHB’s conceptual explanation is *“the background variable of the theory includes the demographic characteristics and includes social influence, previous health care experiences, and environmental resources,”* [7]. Relationship statements are divided into those that express correlation and those that express causality; an example of a correlational statement is *“the interrelationships between cognitive appraisal, intrinsic motivation and affective response are complicated,”* and an example of a causal statement is *“the background variables, which include sociocultural and personal resource, directly influence the individual’s cognitive appraisal”* [7].

### Evaluation of the theory

#### Significance

The significance of a theory is evaluated to check whether concepts and propositions are specified based on philosophical opinions, and whether conceptual models are presented based on philosophical opinions [10]. Cox’s IMCHB is a targeted health promotion theory developed from a holistic point of view of humans and nursing, and aims to induce health behavior through interactions between experts and clients by identifying clients’ background, as well as relevant cognitive and psychological factors. The IMCHB presents three main elements and sub-concepts, and shows their interactions. Cox’s original formulation of the IMCHB did not specify the definition and measurement method of the concepts, but another study by Cox [16] explained each conceptual and operational definition.

#### Internal consistency

Internal consistency ensures that the elements of a theory, the meaning and clarity of the concepts, and terms and definitions are consistent [10]. In the 11 studies reviewed in this study [17-27], only seven experimental studies included the three major components of the IMCHB [18,20,22-25,27,]. All the dynamic variables included in elements of the client’s singularity were displayed in two descriptive studies [21,26] and one experimental study [22]. Of the four studies [21-23,25] that aimed to measure intrinsic motivation, that by Hanrungcharotorn et al. [21] actually measured extrinsic motivations for health behaviors through support from family, friends, and colleagues, but not intrinsic motivations for self-determinants of health behavior within the IMCHB. In seven experimental studies, the elements of client-professional interaction were included, but in two studies, the conceptual meaning of “decisional control” and “professional technical competencies” were unclear. The relationships among the variables of client-professional interactions, such as affective support, health information, decision control, and professional/technical competencies, were also unclear.

#### Parsimony

The parsimony of a theory refers to whether phenomena are clearly explained using as few concepts and propositions as possible. Cox’s IMCHB includes three main elements and a total of 16 sub-concepts. The relationships among the elements and among the concepts are shown in Figure 2. Among the reviewed studies, none measured all 16 sub-concepts, and only three measured affective response, motivation, and cognitive appraisal, corresponding to the dynamic variables affecting health outcomes [21,22,26]. In two studies [18,22] that presented a theoretical framework, the interactions between client’s background variables, such as demographic characteristics, social influence, and previous health care experiences, were not specified.

#### Generality

The generality of a theory refers to whether it is applicable to various population groups [11]. The participants of the reviewed studies were women of various groups, including minorities (immigrants or multicultural women) and low-income women [17,19,23,24,26,], pregnant women [22,25], postpartum women [27], women with gynecological cancer [18], women with osteoarthritis [21], and older adults [20], suggesting adequate generality.

#### Testability

Testability refers to whether the scientific usefulness of a theory can be empirically tested. It can be evaluated in terms of whether research methodology reflects theory, whether there are scales to measure observable concepts, and whether statistical analysis can measure its propositions [10].

In seven studies (63.6%), nursing interventions were provided to the participants, reflecting the specificity of the nursing prescriptions presented in the IMCHB. The main health outcomes measured in the 11 studies were health status [17,22], problem-severity indicators [18,20,21,23,25], adherence to the recommended care regimen [22,24-26], and satisfaction with care [27]. The reviewed studies measured concepts using different scales for each. In two studies, depression status was measured using the Center for Epidemiologic Studies Depression Scale, and pain was measured using a visual analog scale.

The studies used the following statistical methods: the chi-square test; the t-test; analysis of variance (ANOVA), analysis of covariance, and repeated-measures ANOVA; the Mann-Whitney U-test; the Kruskal-Wallis test; correlation analysis; regression analysis; and survival analysis.

#### Empirical adequacy

To evaluate the empirical adequacy of a theory is to analyze whether the theory is consistent with the empirical assertions derived from the research [10]. In the reviewed studies, health outcomes were confirmed according to the variables of client singularity, which are elements of the IMCHB (Table 3). Of the seven experimental studies, six studies based on the IMCHB [18,20,22-25] provided interventions that considered the clients’ background variables and improved health outcomes through client-professional interactions. Cox [16] claimed that previous health outcomes could provide feedback regarding a client’s background variables. In Ackerson’s study [17], previous experiences of Pap smears in minority women were an important factor in receiving regular cervical cancer screening tests. In addition, affective response, intrinsic motivation, and cognitive appraisal—as dynamic variables of the client—were partially supported, with positive effects on health outcomes.

#### Pragmatic adequacy

A theory’s pragmatic adequacy is evaluated using the criteria of (a) the education and training required before utilization in nursing practice, (b) the theory’s application in real nursing practice, (c) the feasibility of implementing theoretical activities, and (d) the professional’s legal capacity to implement the activities [10]. In the reviewed studies, the IMCHB was applied to women’s health promotion in a variety of nursing practices, such as cervical cancer screening, postpartum management, breastfeeding, and physical activity enhancement. These were studies conducted in real-world clinical settings and helped solve the participants’ health problems. In the study of An et al. [18], when caring for gynecological cancer patients, nurses encouraged physical activity, imparted nutritional education to the participants, and stimulated an affective response. Based on these results, it can be concluded that Cox’s IMCHB has excellent pragmatic adequacy.

## Discussion

In this study, the structural and functional elements of Cox’s IMCHB were confirmed through a review of articles on women’s health and the application of the IMCHB, based on the criteria of Fawcett [10] and Chinn and Kramer [11]. Moreover, based on the results of the evaluation, this study aimed to present certain directions to be considered when providing and evaluating nursing interventions that apply the IMCHB in future women’s health research.

First published in 1982, Cox’s IMCHB [7] presents a comprehensive framework in nursing practice, education, and research, including the metaparadigm of nursing. It also emphasizes the philosophical assumptions of nursing from a holistic point of view [28], and underscores the need to assess health from a multidimensional perspective, taking into account the physical, cognitive, and psychological aspects of clients. The IMCHB is special in acknowledging the individual’s singular identity and the right to select health behaviors for their health promotion. It also presents a nursing metaparadigm, furnishes a theoretical framework for evaluating nursing intervention, provides professional/practical health promotion guidelines for nurses, and has contributed to the expansion of knowledge in nursing research. In addition, upon reviewing 11 studies, it was found that the IMCHB has been used as a health promotion intervention strategy for various populations of women and has led to useful results in nursing practice; therefore, it is a suitable theory for experimental and clinical research.

The meanings of the sub-concepts of the three elements of the IMCHB were not unified in each study and were unclear, so internal consistency was somewhat insufficient in the studies included in this review. Cox’s IMCHB includes many concepts and complicated relationships, and therefore lacks clarity and parsimony.

The IMCHB was developed with the goal of devising the best interventions possible by focusing on the interrelationship between clients and nurses [7]. Cox [16] claimed that the IMCHB can guide explanatory and intervention studies for health behavior. This review confirmed that the IMCHB has been applied to suggest directions for nursing interventions and has been helpful for improving health behavior, with positive effects on health outcomes.

After developing the IMCHB, Cox [15] developed the health self-determinism index to systematically measure various intrinsic motivations for health behavior. However, the health self-determinism index was not used in the reviewed literature; on the contrary, in one study, intrinsic motivation was incorrectly measured in terms of extrinsic motivation. This indicates the need for a clear analysis and understanding of the theory and its concepts before applying it to research. No studies measured all five health outcomes or the utilization of healthcare services.

The development of the IMCHB started with the aim of developing a theory of health behavior that can be practically used from the nursing perspective [7]. Accordingly, this review confirmed that IMCHB has clearly guided practice and research to provide appropriate nursing for patients. Also, as a high-level prescribing theory, the IMCHB can be helpful in nursing research and practice to develop further knowledge in the field of nursing [29].

In summary, this review revealed that the IMCHB has significance, generality, testability, empirical adequacy, and pragmatic adequacy for nursing practice and research. However, the lack of clear conceptual definitions and the complex relationships among concepts result in a lack of internal consistency and parsimony. As such, the IMCHB is a suitable theory for guiding practice and research in women’s health promotion and the development and application of interventions. Future research can build on this middle-range theory in women’s health research and practice.

## Figures and Tables

**Table 1.** Characteristics of studies (n=11)

Variable	Categories	n (%)
Country	International	5 (45.5)
	South Korea	6 (54.5)
Research design	Quantitative	10 (90.9)
	Non-experimental design: Survey	3 (27.3)
	Experimental design: Quasi-experimental	7 (63.6)
	Qualitative	1 (9.1)
	Content analysis	1 (9.1)
Population	Minority and low-income women	5 (45.5)
	Pregnant women	2 (18.2)
	Postpartum women	1 (9.1)
	Women with osteoarthritis	1 (9.1)
	Women with gynecological cancer	1 (9.1)
	Older adults	1 (9.1)

**Table 2.** Conceptual definition of elements and variables

Element and variable	Definition
Client singularity	Unique intrapersonal and contextual configuration of an individual based on background variables, motivation, cognitive appraisal, and affective response
Background variables	Relatively non-modifiable influences on health behavior
Demographic characteristics	Client characteristics: age, education, race
Social influence	Social factors that affect health behaviors
Previous healthcare experience	Health history (objective and subjective), current physiological health status, and developmental status
Environmental resources	Availability of informational, people, financial, and geographic resources to facilitate health behavior
Dynamic variables	Modifiable targets for intervention
Affective response	Emotional response to a health concern
Motivation	Intrinsic or extrinsic motivation, self-determination
Cognitive appraisal	Cognitive representation of a health concern
Client-professional interaction	The extent to which the provider attends to a client’s singularity and tailors the intervention approach to that singularity
Affective support	The process of attending to a client’s level of emotional arousal and building an affiliative bond with the client
Provision of health information	The process of providing useful health information to a client
Decisional control	The process of creating a health care climate that is supportive of autonomy rather than controlling
Professional or technical competencies	Therapeutic skills of the provider
Health outcomes	Health behavior or health state that is behaviorally related
Healthcare utilization	The extent to which an individual seeks and uses available health care resources
Health status indicators	Physiological, psychological, social health, and wellbeing parameters
Problem-severity indicators	Disease progression, stabilization as a function of measures of disease or treatment sequelae
Adherence to the recommended care regimen	Extent to which a patient engages in care regimens, behaviors, or treatments that are necessary to ensure optimal health
Satisfaction with care	Client’s appraisal of adequacy of a provider’s response to a health care problem and extent to which the patient’s expectations are met/unmet

**Table 3.** Studies guided by the interaction model of client health behavior

Author, year/ Country	Purpose	Participants	Concept measure	Analytic technique	Main results
Ackerson., 2011 [17]/USA	To explore African- American women’s use of Pap smear	11 women with routine examination (Pap smear) and 13 women with non-routine examination	-Client singularity: age, level of education, income, insurance	Content analysis	The routine and non-routine examination group fit the IMCHB model. Non-routine examination women have negative personal influence.
			-Outcome: personal influencing factors (social influence, previous health care experience, cognitive appraisal) that contribute to Pap smear testing		
An et al.,2015 [18]/South Korea	To examine the effect of lifestyle intervention on the development of fatigue, nutritional status and quality of life of patients with gynecological cancer patients	49 patients with gynecologic cancer patients (E:24; C:25)	-Client singularity: Age, marriage, education, diagnosis, stage, disease period, BMI, body weight, income, fatigue, nutritional states, quality of life	χ^2^-test, t-test, ANCOVA	Lifestyle intervention was effective in lessening fatigue, and improving nutritional status and social/family well-being
			-Outcome: FACIT-F, PG-SGA, FACT-G		
Choi et al., 2011 [19]/USA	To investigate the patterns of physical activity and demographic characteristics with those patterns in Korean immigrants in the USA	197 women Korean immigrants in the USA	-Client singularity: Age, education, employment, income, marital status, having children	χ^2^-test, Mann-Whitney tests, ANOVA, Kruskal-Wallis test,	There was a difference in pattern and amount of activity according to demographic variables (job type, marital status, age, transportation).
			-Outcome: Physical activity (International physical activity questionnaire)		
Chun et al., 2017 [20] /South Korea	To examine the effectiveness of a sleep improvement program combined with an aroma-necklace for elderly women	70 elderly women (E:35; C:35)	-Client singularity: age, religion	χ^2^-test, t-test, Mann-Whitney test	Group differences were found in sleep quality, sleep duration, sleep satisfaction, depression, and anxiety.
			- Outcome: CES-D, state trait anxiety inventory- sleep quality, sleep duration, VAS to measure the sleep satisfaction, blood pressure		No differences were found in the systolic or diastolic blood pressure
Hanrungcharotorn et al. 2017 [21]/Thailand	To examine the factors influencing physical activity among women with osteoarthritis of the knee	242 females with osteoarthritis of the knee attending the outpatient	-Client singularity: age, occupational/marital status, education, income, living status, BMI, medical records, onset of diagnosis, comorbidity	Binary logistic regression, multivariate logistic regression, Pearson correlation	BMI and pain-related fear influenced physical activity. Age, knee pain, functional limitation, pain catastrophizing, and social support did not significantly influence physical activity
			- Outcome: Revised Thai-WOMAC, rMSPSS, CSCSQ, FABQ- PA		
Kim & Kim., 2013 [22]/South Korea	To examine the effect of an integrated self-management program on self- management, glycemic control, and maternal identity in women with GDM	55 women with GDM	-Client singularity: Knowledge of GDM, Self-efficacy of GDM management.	χ^2^-test, Mann-Whitney U test	Integrated self-management program for women with GDM improves self-management, maternal identity, and decreased glycose level.
			-Outcome: Self-management, maternal identify, 2-hour postprandial glucose levels		There was no statistically significant effect in Hb A1c.
Lee et al, 2017 [23]/South Korea	To examine the long-term effectiveness of stretching exercises on the health outcomes of Korean-	80 Korean-Chinese female migrant workers	-Client-professional interaction: Exercise adherence	χ^2^-test, t-test, linear mixed model	Community-based stretching program was effective in increasing their flexibility and decreasing work-related musculoskeletal disorder symptoms
	Chinese female migrant workers		-Outcome: Musculoskeletal fitness, musculoskeletal symptoms, acculturative stress		
Park & Choi, 2014 [24]/South Korea	To examine reproductive health programs for the improvement of reproductive health of female immigrants and to verify their effectiveness.	58 female immigrants in South Korea (E:29; C:29)	-Client singularity: Reproductive health knowledge, reproductive health attitude	χ^2^-test, t-test	Reproductive health program had improved aspects of reproductive health knowledge, reproductive health attitude, and reproductive health behaviors. There were no significant differences in clinical indicators (CBC, urine analysis, vaginal smear) between the experiment group and the control group
			-Outcome: Reproductive health behavior, clinical indicators (CBC, urine analysis, vaginal smear)		
Park & Lee., 2018 [25] /South Korea	To compare the effectiveness of the oral health program and walking exercise program for pregnant women	65 pregnant women (oral health program :23; walking exercise program: 21; C: 21)	- Outcome: Oral health behaviors, nutrition management, international physical activity questionnaire, periodontal disease, CES-D, perceived stress, maternal stress, quality of life related to health (SF-12)	χ^2^ -test, Fisher’s exact test, ANOVA, repeated measures ANOVA	Oral health program and walking exercise program was effective improvements in oral health behaviors, periodontal disease, and psychological indicators as compared to the control group.
					
Tenfelde et al., 2012 [26]/USA	To examine the factors that affect breastfeeding cessation in low-income mothers	309 low income breast- feeding participants with special supplemental nutrient program for women	-Client singularity: Age, education, marital status, ethnicity, knowledge of breast-feeding, breast- feeding support, cognitive, motivational, and affective intrapersonal variables	Survival analysis	Risks factors of breastfeeding cessation were younger, not from Mexican descent, and had no breast-feeding support system.
			-Outcome: Breastfeeding cessation,		
Wagner et al., 2011 [27]/USA	To investigate the impact of nurses' discharge method on postpartum care	70 postpartum women in the USA	-Outcome: Self-administered modified client satisfaction tool	χ^2^-test, Mann-Whitney U test	There is no difference in satisfaction with treatment between demonstration/return demonstration and traditional methods.

ANOVA: analysis of variance; ANCOVA: analysis of covariance; BMI: body mass index; C: control group; CBC: complete blood count; CES-D: Center for Epidemiologic Studies Depression Scale; CSCSQ: the Thai version of the Catastrophizing Subscale of the Coping Strategies Questionnaire; E: experimental group; FABQ-PA: the Thai version of the Fear-Avoidance Beliefs Questionnaire about Physical Activity; FACIT-F: Functional Assessment of Chronic Illness Therapy-Fatigue Scale; FACT-G: Functional Assessment of Cancer Therapy-General; GDM : gestational diabetes mellitus; HbA1c: hemoglobin A1c; IMCHB: interaction model of client health behavior; Pap: Papanicolaou; PG-SGA: patient-generated subjective global assessment; rMSPSS: the revised Thai Multi-Dimensional scale of Perceived Social Support; SF-12: Short Form 12-item; Thai-WOMAC: Thai version of the Western Ontario and McMaster Universities Osteoarthritis Index; VAS: visual analog scale.
